# Neurodevelopmental outcomes following possible serious bacterial infection in early infancy in Karachi, Pakistan: a prospective cohort study

**DOI:** 10.1186/s12887-024-04780-5

**Published:** 2024-05-15

**Authors:** Nudrat Farheen, Shahira Shahid, Kiran Ramzan Ali Lalani, Iqbal Azam, Farah Khalid, Batool Fatima, Mohammad Shahidul Islam, Samir K. Saha, Shamim Ahmad Qazi, Fyezah Jehan, Muhammad Imran Nisar

**Affiliations:** 1 Department of Pediatrics and Child Health, Aga Khan University, Stadium Road, Karachi, 74800 Pakistan; 2 Child Health Research Foundation, Dhaka, Bangladesh; 3 Independent Consultant, Geneva, Switzerland; 4 Department of Community Health Sciences, Aga Khan University, Karachi, Pakistan

**Keywords:** Neurodevelopment, Newborns, Young infants, Possible serious bacterial infection, Pakistan, Childhood

## Abstract

**Background:**

Pakistan reports a significant burden of neonatal mortality, with infections as one of the major causes. We aim to assess the long-term impact of early infancy infections on neurodevelopmental outcomes during later childhood.

**Methods:**

We conducted a prospective follow-up study of the cohort enrolled at the Karachi site of the Aetiology of Neonatal Infection in South Asia (ANISA) during 2019–2020. Children with a possible serious bacterial infection (based on the WHO IMCI algorithm) at early infancy were assessed for neurodevelopment at 6–9 years of age and compared with healthy controls. The Ten Questions (TQS) questionnaire, Strengths and Difficulties Questionnaire (SDQ), and Parent’s Evaluation of Developmental Stage Assessment Level (PEDS: DM-AL) neurodevelopmental assessment tools, were administered and scored by the research staff who were blinded to the child’s exposure status. Generalized Structural Equation Modelling (GSEM) was employed to verify relationships and associations among developmental milestones, anthropometry, and sociodemographic variables.

**Results:**

A total of 398 children (241 cases and 157 controls) completed neurodevelopmental and growth assessments. Cases had a significantly higher rate of abnormal TQS scores (54.5% vs. 35.0%, *p*-value 0.001), greater delays in motor milestones (21.2% vs. 12.1%, *p*-value 0.02), lower fine motor skills (78.4 ± 1.4 vs. 83.2 ± 1.5, *p*-value 0.02). The receptive language skills were well-developed in both groups. According to the logistic regression model, exposure to infection during the first 59 days of life was associated with delayed TQS milestones (β = -0.6, 95% CI -1.2,-0.04), TQS hearing domain (β = -0.3, 95% CI: -1.2 to 0.7), PEDS: DM-AL fine motor domain (β = -1.3, 95% CI: -4.4 to 1.7), PEDS: DM-AL receptive language development (β = -1.1, 95% CI: -3.7 to 1.4) and child anthropometric measurements such as weight and height (β = -0.2, 95% CI: -0.4 to 0.01 and β = -0.2, 95% CI: -0.4 to -0.01, respectively). Early pSBI exposure was positively associated with PEDS: DM-AL self-help domain (β = 0.6, 95% CI: -1.2 to 2.4) and SDQ-P overall score (β = 0.02, 95% CI: -0.3 to 0.3).

**Conclusion:**

Children exposed to PSBI during early infancy have higher rates of abnormal development, motor delays, and lower fine motor skills during later childhood in Pakistan. Socioeconomic challenges and limited healthcare access contribute to these challenges, highlighting the need for long-term follow-ups with integrated neurodevelopment assessments.

**Supplementary Information:**

The online version contains supplementary material available at 10.1186/s12887-024-04780-5.

## Introduction

Despite the tremendous progress made over the last two decades in improving newborn health, globally, around 2.4 million newborns die before completing their first month of life [1]. The majority (79%) of these deaths occur in Sub-Saharan Africa and South Asia [2]. Pakistan has one of the highest Neonatal Mortality Rate (NMR) in the world at 39.4 deaths per 1000 live births [1]. An annual birth cohort of 5.5 million translates to potentially 251,000 neonates deaths per year in Pakistan. Infections remain one of the top most causes of mortality in this age group [1]. During 2009–2018, the global incidence of neonatal infections was estimated to be 3930 episodes per 100,000 live births (95% CI 1,937-7,812) [3]. A prospective, community-based multicenter study, the Aetiology of Newborn Infections in South Asia study (ANISA), estimated the incidence of Possible Serious Bacterial Infection (pSBI) among young infants to be 95.4 (95% CI 78.3-112.6) per 1000 live births [4].

While it is well established that infections cause significant mortality, their long-term neurodevelopmental outcomes are not well-documented. It has been proposed that systemic inflammation and hypoxic-ischemic injury, which occur upstream at the site of infection instigate a cascade of processes leading to brain injury and a decline in nutritional supply to the brain [5]. According to the Global Burden of Disease study, an estimated 52.9 million (8.4%) children under the age of five years worldwide have a developmental disability and 94.9% of these children are from Low- and Middle-Income Countries (LMICs) [6]. Early screening and intervention during critical periods of neurodevelopment in children can partially or completely reverse adverse outcomes [7].

The ANISA study identified the etiology of pSBI in young infants less than two months of age at community level in three South Asian countries i.e., Bangladesh, India, and Pakistan [4]. In this study, we follow up on the cases and controls from the ANISA Pakistan cohort at age 6–9 years to compare neurodevelopmental outcomes in the two groups. We also describe predictors for adverse neurodevelopmental outcomes in these children.

## Methods

### Study design and setting

The ANISA study was done in five sites across three countries in South Asia- Bangladesh, Pakistan, and India. A pregnancy cohort of 74,145 women at these sites gave birth to 71,361 liveborn during the study period. These newborns were followed for the first 2 months of life by trained Community Health Workers (CHWs) with ten home visits and assessed for signs of severe illness using the WHO Integrated Management of Childhood Illness (IMCI) 7-sign algorithm. A pSBI case was defined as a young infant aged 0–59 days presenting with any of the following signs: fast breathing (respiratory rate ≥ 60 per minute), severe chest indrawing, no movement at all or movement only when stimulated, not able to feed at all or not feeding well/stopped feeding well, convulsions, high body temperature (≥ 38° C) and low body temperature (< 35.5° C) [8]. Among pSBI cases enrolled from Karachi, 40% had rapid breathing (≥ 60 breaths per minute), 26% showed severe chest in-drawing, 31% had a high axillary temperature (≥ 38·0 °C), 13% had a low axillary temperature (< 35·5 °C), 11% showed movement only when stimulated or no movement, and 6% had a history of convulsions or observed convulsions. Additionally, 37% experienced poor feeding, and 51% showed more than one of these signs. Suspected cases of clinical infection were referred to study clinics or hospitals, for confirmation by a physician and blood, and respiratory samples were collected, and antibiotics were prescribed. Only 15% (*n* = 15) of the blood samples of pSBI cases were positive on culture. In addition, age and gender matched healthy controls were identified from the cohort and blood and respiratory samples were collected. Detailed methodology has been published previously [4, 9, 10].

We enrolled a subset of pSBI cases (young infants with clinical signs of PSBI according to WHO IMCI [8] and healthy controls (Healthy controls were infants with no signs of pSBI at any of the CHW visits until 2 months of age) from the ANISA study enrolled between January 2012 and December 2013 in Karachi, Pakistan and assessed their growth and neurodevelopment from November 2019-October 2020. Follow-up activities were conducted in four out of the five colonies where the ANISA study was carried out, namely Ibrahim Hyderi, Ali Akber Shah Goth, Rehri Goth, and Bhains Colony. We included all cases and controls residing in the study area, obtaining written consent from caregivers of study participants. Assent was obtained from all participants aged 7 years or older who consented to be part of the study. Our exclusion criteria comprised children with known adverse neurodevelopmental outcomes, such as cerebral palsy, inborn errors of metabolism, gross congenital malformations, or neurological disabilities. Additionally, we excluded cases of loss to follow-ups, deaths, and refusals to consent [4].

A team of trained psychologists and community health workers (CHWs), blinded to the case-control status, performed household visits for data collection. Written informed consent was obtained from caregivers of the study participants and assent was taken from all participants of age seven years or older. A questionnaire was administered to the caregiver which covered socioeconomic and demographic features of the household; maternal characteristics like history of addictions, physical and psychological trauma; and child characteristics like birthweight, gestational age, history of breastfeeding and history of recent infections. Anthropometric measurements of the children were taken using the SECA (model 874, Columbia, MD, USA) machine for weight, a calibrated measurement tape for height, and UNICEF-S0145620 tape for mid-upper arm circumference (MUAC). Age-standardized Z-scores were calculated for weight, height, and BMI using WHO references [11].

For neurodevelopment assessment, we used the validated Urdu translation of the Ten Questions (TQS) questionnaire. It comprises 10 questions with binary responses on a child’s cognitive and motor impairment, seizure disorders, speech, vision, and hearing impairments [12]. We also used the validated Urdu version of the 25-item Strength and Difficulties Questionnaire (SDQ-P) covering 5 domains with 5 items each rated on a 3-point Likert scale, 0 for not true, 1 for somewhat true and 2 for certainly true [13]. Additionally, we used a validated version of the Parents Evaluation of Developmental Stages-Developmental Milestones-Assessment Level (PEDS: DM-AL) tool. It provides age-equivalent and the percentage of delay scores as well as the percentage of skills mastered in various domains such as fine motor, gross motor, expressive language, receptive language, self-help, social-emotional, academic/pre-academic, and cognitive [14].

All data was collected on tablets using electronic questionnaires except for the PEDS: DM-AL tool, which was collected on paper forms and entered using RedCap software.

A sample size of 269 each for cases and controls was calculated assuming twice the risk of adverse neurodevelopmental outcome at 6–9 years of age in the pSBI group compared to the healthy controls, with background rates of 13.6% [15]. A two-sided alpha of 5% with 80% power was used. We carried out a pilot study for 10% of the sample size.

### Analysis

A positive TQS test is defined as a positive response to any one of the 10 questions [12]. An SDQ-P score of 17 and above is considered abnormal [13]. The PEDS: DM-AL abnormal and borderline scoring ranges are defined as 0–15% and > 15-25% skills attained according to age, respectively [14].

Descriptive statistics were reported using frequencies and percentages for categorical variables and mean ± SD for continuous variables. Crude estimation was conducted using chi-square and t-tests with a significance level of 5%. We used the generalized structural equation model (GSEM) to determine factors associated with each domain of neurodevelopment and anthropometry. Each domain of TQS and MUAC was a binary variable analyzed using a logit link function. SDQ score was a latent variable, and the remaining variables (weight, height, fine motor, self-help, receptive language, and expressive language) were analyzed using the identity link function. Pathways for each variable that was significant at an alpha of 0.25 were included in the multivariable model, which was developed using a forward stepwise approach. We applied Akaike’s information criterion (AIC)-informed model selection to select the best-fitting model based on minimizing the model’s AIC. Models with a decrease in AIC of at least 2 units per degree of freedom were favored during the model building. Multicollinearity was checked for all variables. Statistically significant effects were assumed for *p*-value < 0.05 at a confidence interval of 95%. We conducted statistical analyses using Stata 16.0 software.

### Ethical statement

Ethical approval was taken from the Ethical Review Committee (ERC no. 2021-3415-17061) of Aga Khan University (AKU) after clearance from the Departmental Review Committee of Community Health Sciences. Consent forms were filled out for mothers in their preferred language. When significant developmental delays were identified by study staff, mothers and children were referred for further assessment and management.

## Results

**Fig. 1:**
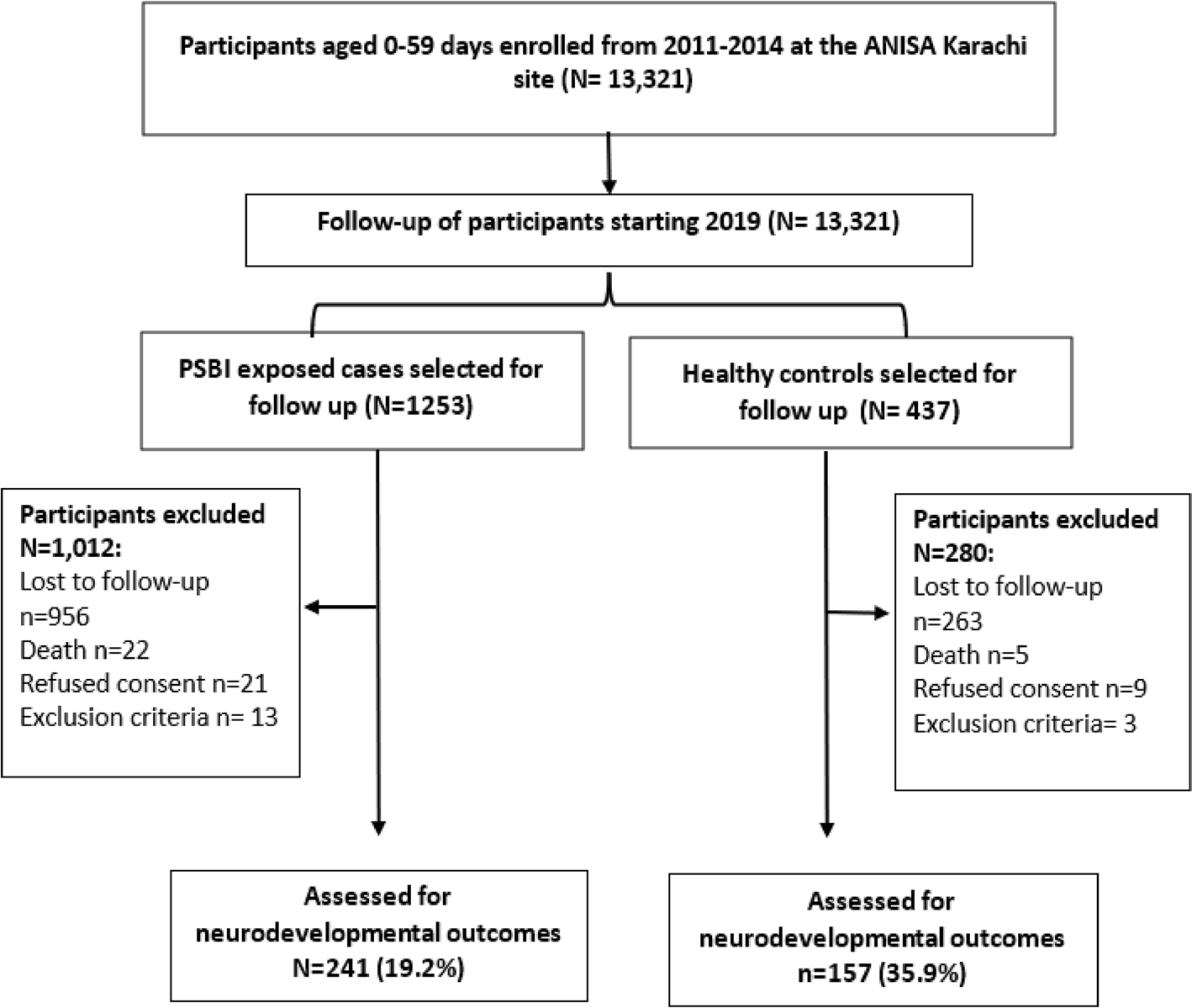
Flowchart showing enrollment of participants in the study

Between 2012 and 2013, a total of 13,321 children aged 0-59 days were enrolled in the ANISA Pakistan cohort. Among them, 1,253 cases and 437 controls were followed up from 2019-2020. We excluded 1,012 cases and 289 controls from the study because they did not meet the inclusion criteria. A majority of the excluded children (843 cases and 132 controls) had migrated out of the study area before the follow-up assessments were conducted and could not be reached. Thus, neurodevelopmental assessments could only be conducted on 240 (19.2%) cases and 157 (35.9%) controls, respectively. Figure 1 describes the flow of participants in the study.

### Sociodemographic and anthropometric characteristics

Table 1 describes the sociodemographic characteristics of the study groups. The Ibrahim Hyderi study site demonstrated a significantly higher proportion of pSBI cases (*p*=0.02). Around two-thirds of the participants, (75.1%) cases and (66.2%) of controls, belonged to the 6 to 7 years age group. A higher percentage of males were observed among pSBI cases (57.3%) compared to the control group (44.6%) (*p*=0.01). PSBI cases had a higher number of deaths among siblings as compared to the control group (*p*=0.02) and required more time to reach the nearest clinic or hospital than controls (*p*=0.03). Additionally, pSBI cases exhibited a lower frequency of exclusive breastfeeding for 6 months compared to the control group (*p*=0.03).

**Table 1:** Baseline characteristics of the study participants

Characteristics	PSBI cases (*n* = 241)	Control group (*n* = 157)	*P*-value
	Freq (%); Mean ± SD	Freq (%); Mean ± SD	
**Demographic factors**			
Field site			
-Ibrahim Hyderi	99(41.1%)	59(37.6%)	0.02
-Rehri goth	66(27.4%)	27(17.2%)	
-Ali Akber Shah goth	28(11.6%)	28(17.8%)	
-Bhains colony	48(19.9%)	43(27.4%)	
Age of study participant (in months)	83.1 ± 4.8	83.9 ± 5.8	0.14
Age groups			
- 6 to 7 years old	181(75.1%)	104(66.2%)	0.06
- Older than 7 years	60(25.0%)	53(33.8%)	
Gender			
-Male	138(57.3%)	70(44.6%)	0.01
-Female	103(42.7%)	87(55.4%)	
Age of mother (in years)	32.7 ± 5.6	32.8 ± 5.9	0.90
Age of father (in years)	37.4 ± 5.4	37.9 ± 7.0	0.40
Consanguineous marriage	158(65.6%)	91(58.0%)	0.10
Number of siblings alive	4.7 ± 2.3	4.6 ± 2.2	0.70
Number of siblings who died	0.7 ± 1.1	0.5 ± 0.9	0.02
**Socioeconomic factors**			
Ever registered in school	144(59.8%)	99(63.1%)	0.50
Ability to read/write any language	65(27.0%)	36(22.9%)	0.06
Father currently employed	228(94.6%)	143(91.1%)	0.30
Mother currently employed	48(20%)	39(24.8%)	0.20
Residence			
-Own	134(85.4%)	134(85.4%)	0.009
-Rent	23(14.6%)	23(14.6%)	
Members living in same household	8.6 ± 4.1	8.6 ± 4.1	
Socioeconomic status^a^			
-Least privileged	70 (29.0%)	36(22.9%)	
-Lesser privileged -Fair privileged -Better privileged	60(24.9%) 65(27.0%) 46(19.1%)	38(24.2%) 40(25.5%) 43(27.4%)	0.20
Fuel used for cooking			
-Wood	52(21.6%)	24(15.3%)	0.1
-Gas	189(78.4%)	133(84.7%)	
Water for drinking			
-Tap water	163(67.3%)	98(62.4%)	0.2
-Other (Filtered or mineral)	78(32.4%)	59(37.6%)	
Time to reach nearest clinic/hospital (minutes^b^)	18.8 ± 13.8	15.9 ± 12.4	0.03
**Addiction/abuse/trauma**			
Exposure to indirect smoke (huqqa/ smoking)^c^	25(10.4%)	22(14.0%)	0.6
Study participant addiction^d^,	9(2.3%)	4(1.0%)	0.4
Caregiver reported physical abuse to study participants^e^	17(7.1%)	11(7.0%)	1.0
Mental trauma			
-None	71(29.5%)	56(35.7%)	0.9
-Any death in family	28 (11.6%)	16 (10.2%)	
-Parental separation	9 (3.7%)	6 (3.8%)	
-Fights among parents	85 (35.3%)	45 (28.7%)	
-Bullied at home/school	48 (19.9%)	34 (21.7%)	
**Maternal characteristics**			
Chronic illness during pregnancy			
-None	212(88.0%)	133(84.7%)	0.4
-HTN	20(8.3%)	22(14.0%)	
-Others^f^	9(3.7%)	2(1.3%)	
Leaking membranes for > 18 hours before delivery	38(15.9%)	22(14.0%)	0.6
Fever in the week prior to delivery	16(6.8%)	10(6.4%)	0.9
Mode of delivery			
-Vaginal	220(91.3%)	136(86.6%)	0.1
-C-section^g^	21(8.7%)	21(13.4%)	
Place of Delivery			
-At home (untrained birth attendant)	18(7.5%)	15(9.6%)	0.2
-At home by Trained Birth Attendant	78(32.4%)	61(38.9%)	
-Facility birth	145(60.2%)	81(51.6%)	
Gestational age at birth			
-Term	163(67.3%)	120(76.4%)	0.06
-Preterm	78(32.4%)	37(23.6%)	
Weight at birth^h^			
-Adequate	218(90.5%)	135(86.0%)	0.2
-Low birth weight	23(9.5%)	22(14.0%)	
**Early nutritional factors**			
Ever breastfed	234(97.1%)	149(94.9%)	0.3
Exclusive breastfeeding for 6 months	119(49.4%)	71(45.2%)	0.03
Non-breast milk given in the first 2 years	118(49.0%)	72(45.9%)	0.6
Age at weaning (months)	6.9 ± 4.8	6.3 ± 3.1	0.09
**Infections**			
Incidence of mild infections in last 6 months^i^			
-At least 2 or more	63(26.1%)	33(21.0%)	0.2
-1 Or none	178(73.9%)	124(79.0%)	
Incidence of severe diarrhea in last 6 months			
-At least 2 or more	14(5.8%)	9(5.7%)	1.0
-1 Or none	227(94.2%)	148(97.3%)	
**Family history**			
ND abnormalities among parents or siblings^j^	24(10.0%)	9(5.7%)	0.1

^a^Division of groups based on index-score made from using common-use household items and transportation (electric fan, simple mobile phone electric iron, television, refrigerator, motorbike, smartphone, computer/laptop, car), adapted from Pakistan social and living standards measurement tool by Pakistan Bureau of Statistics.

^b^Time is reported based on transport which is usually available

^c^Smoke which can cause unavoidable passive addiction among others living in same house due to shared space

^d^Nuswar, chewable tobacco, substance abuse, I/v drugs (126 in infected cases, 76 in controls; majority Nuswar and tobacco eaters); or alcohol (5 in infected cases, 9 in controls) All that apply (alcohol, chewable tobacco, smoking, drugs)

^e^Hard hitting causing bruising/ wounds

^f^Jaundice (4), Anemia (2), TB (1), HIV (1)

^g^Includes elective and emergency c-sections

^h^Reported by caregiver

^i^Diarrhea < 3 days, URTI for < 5days, Ear infection without complications

^j^Presence of any of these among parents or siblings: febrile fits; epilepsy or abnormal movements; very low IQ; mental retardation; visual or speech impairment; delayed milestones; regressed milestones; static milestones; unusual behavior; diagnosed mental disorder

Preterm births were higher among cases (32.4%) compared to the control group (23.6%). The cases also had a higher prevalence of family history of neurodevelopmental disorders (10.0%) compared to the controls (5.7%). In both groups, the majority of children had lower-than-normal height for age, weight for age, and BMI for age (0 to -2 SD). However, these results were not statistically significant. The two groups did not show any significant differences in terms of addiction and psychological trauma history, MUAC (mid-upper arm circumference), or weight and height for age at the time of outcome assessment.

### Neurodevelopmental outcomes

**Fig. 2:**
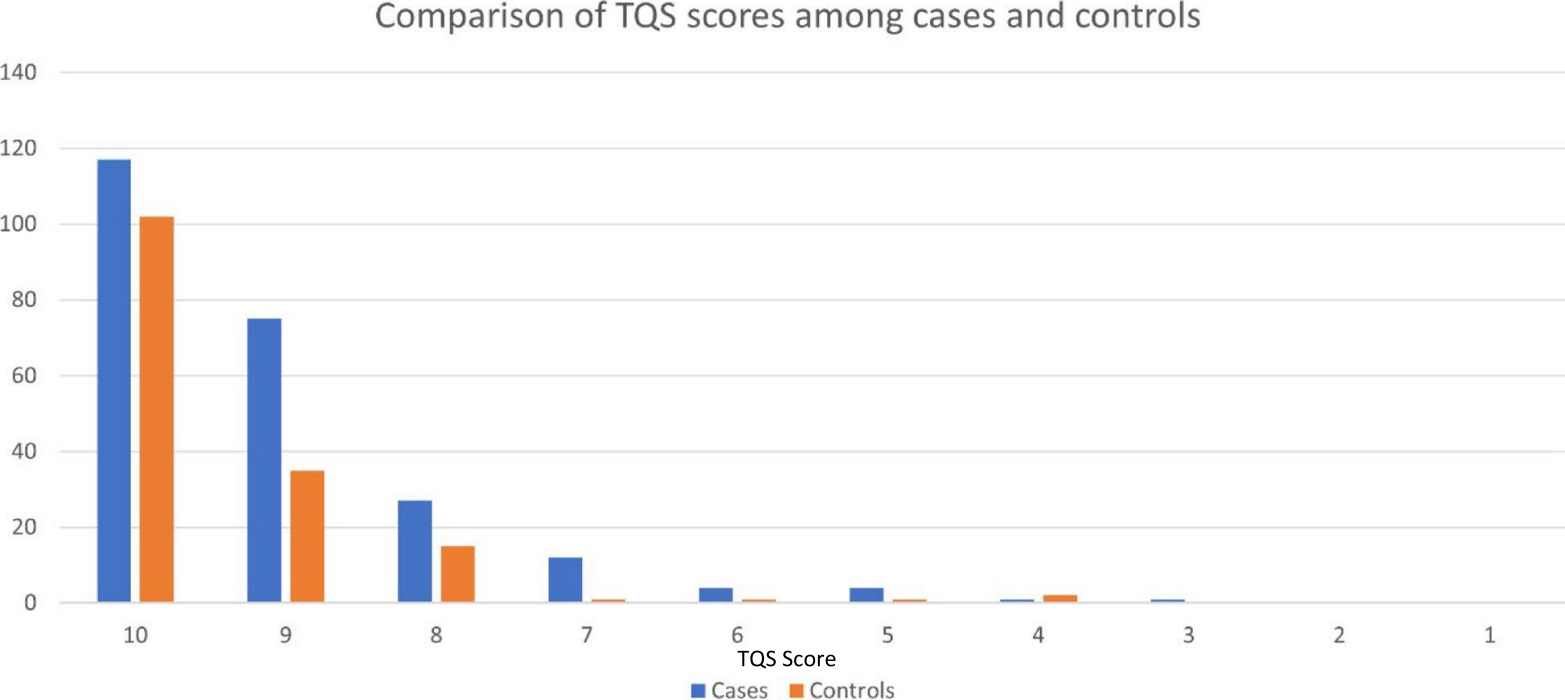
Comparison of TQS scores among cases and controls, *n* = 398

Three hundred ninety-eight children, including 241 cases and 157 controls completed neurodevelopmental testing. Out of these, cases exhibited a significantly higher rate of abnormal TQS scores compared to the controls (54.5% vs. 35.0%, *p*-value 0.001). Cases also demonstrated a greater delay in achieving individual TQS milestones, particularly in motor milestones (21.2% vs. 12.1%, *p*-value 0.02) (Fig. 2 and Table 2).

**Table 2:** Screening results for the Ten Question Screening (TQS) questionnaire

Description	PSBI cases (*n* = 241)	Control group (*n* = 157)	*p*-value
Total (Mean ± SD)	9.1 ± 0.1	9.4 ± 0.1	0.01
Normal (score 10) (%)	117(48.6%)	102(65.0%)	Ref
Abnormal (score 0–9) (%)	124(54.5%)	55(35.0%)	
Motor milestones delay	51(21.2%)	19(12.1%)	0.02
Difficulty in seeing	14(5.8%)	8(5.1%)	0.8
Difficulty in hearing	14(5.8%)	7(4.5%)	0.6
Receptive language problem	15(6.2%)	6(3.8%)	0.3
Current motor issues	24(10.0%)	12(7.6%)	0.4
Fits or fainting	9(3.7%)	6(3.8%)	1.0
Problem in learning skills	15(6.2%)	6(3.8%)	0.9
Problem in recognizing words	15(6.2%)	5(3.2%)	0.2
Problem in speech (different from normal)	24(10.0%)	8(5.1%)	0.08
Mentally backward for age	34(14.1%)	15(9.6)	0.2

When assessing the SDQ scores based on standardized cut-offs, 41.5% of the cases and 33.1% of the controls showed abnormal scores, although the difference was not statistically significant and there were no notable differences in scores across individual domains (Table 3).

**Table 3:** Screening results for the strengths and difficulties questionnaire (SDQ)

Description (Mean ± SD)	PSBI cases (*n* = 241)	Control group (*n* = 157)	*p*-value
Emotional problems score	2.0 ± 0.1	1.8 ± 0.2	0.3
Conduct problems score	2.2 ± 0.1	2.0 ± 0.2	0.3
Hyperactivity problems score	2.0 ± 0.1	1.7 ± 0.2	0.2
Peer problems score	1 ± 0.1	1 ± 0.1	0.9
Prosocial behaviour problems score	9.1 ± 0.1	9.3 ± 0.1	0.3
Total score	16.4 ± 0.4	15.7 ± 0.5	0.3
Abnormal (%)	100 (41.5%)	52 (33.1%)	0.09

**Fig. 3:**
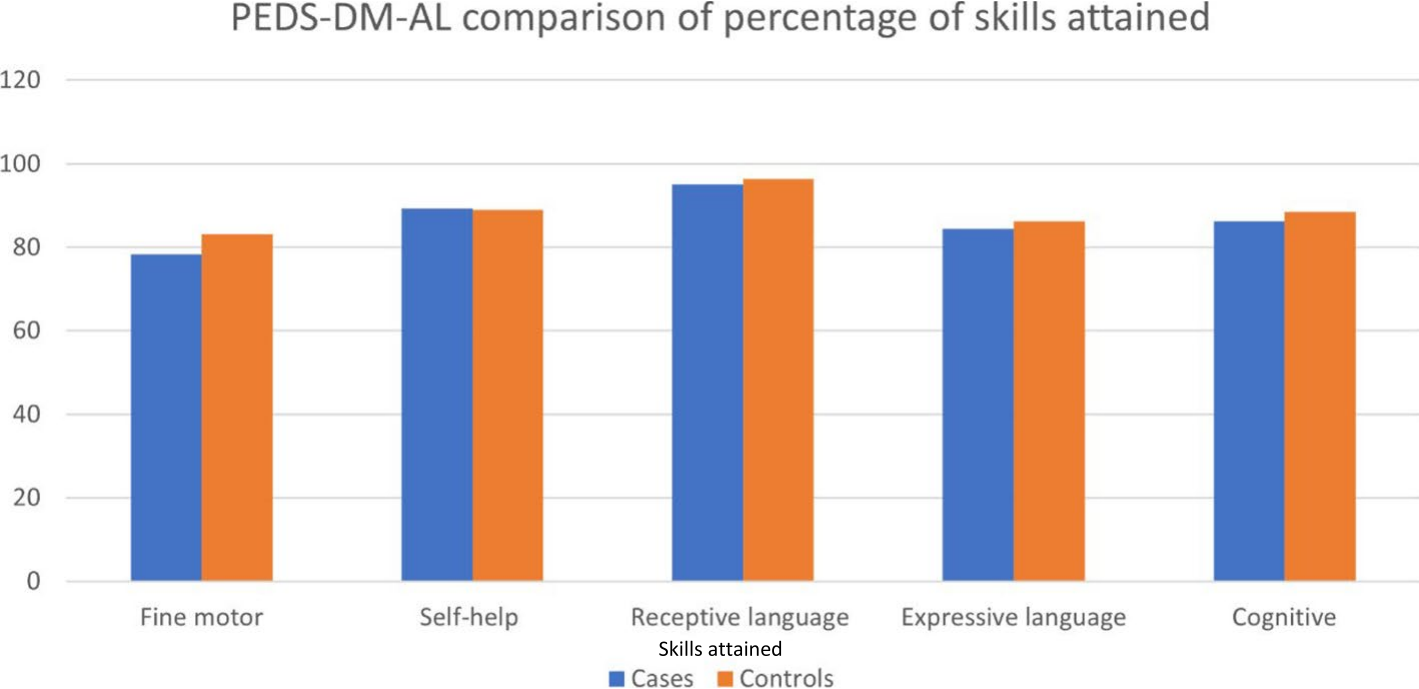
Parents’ evaluation of developmental staging-developmental milestones-assessment level form, *n* = 398

According to the PEDS:DM-AL tool, the cases exhibited significantly lower attainment of fine motor skills compared to the control group (78.4 ± 1.4 vs. 83.2 ± 1.5, *p*-value 0.02). Receptive language skills were the most well-developed among both groups, with approximately 95% of children achieving the best skill level in this area (Fig. 3 and Table 4).

**Table 4:** (PEDS:DM-AL)-comparison of the percentage of skills attained according to age

Item Description (Mean ± SD)	PSBI cases (*n* = 241)	Control group (*n* = 157)	*p*-value
Fine motor, % of skills attained	78.4 ± 1.4	83.2 ± 1.5	0.02
Self-help, % of skills attained	89.3 ± 0.6	89.0 ± 0.7	0.8
Receptive language, % of skills attained	95.2 ± 0.9	96.4 ± 1.0	0.4
Expressive language, % of skills attained	84.5 ± 0.8	86.2 ± 0.8	0.1
Cognitive, % of skills attained	86.7 ± 0.8	88.6 ± 0.8	0.07

### Factors associated with neurodevelopment and growth

In the multivariable GSEM, exposure to infection during 0-59 days of life (β = -0.6, 95% CI -1.2,-0.04) was significantly associated with delayed TQS milestones. pSBI exposure was negatively associated with TQS associated hearing domain (β = -0.3, 95% CI: -1.2 to 0.7) However, it was not statistically significant.

pSBI exposure was positively associated with SDQ overall score (β = 0.02, 95% CI: -0.3 to 0.3). The PEDS:DM-AL fine motor domain was negatively associated with pSBI cases (β =: -1.3, 95% CI: -4.4 to 1.7). receptive language development showed negative association (β = -1.1, 95% CI: -3.7 to 1.4) whereas the PEDS:DM-AL self-help demonstrated a positive association (β =: 0.6, 95% CI: -1.2 to 2.4). Although none of these were statistically significant.

Exposure to infection was significantly associated with anthropometric measurements such as weight and height of the child (β = -0.2, 95% CI: -0.4 to 0.01) and (β = -0.2, 95% CI: -0.4 to -0.01) respectively. Other variables associated with child neurodevelopment are shown in Table 5.

**Table 5:** Factors associated with neurodevelopmental and anthropometric outcomes according to the GSEM model

Outcomes	TQS			SDQ	PEDS: DM-AL				Anthropometry		
	Milestones	Vision	Hearing	Total score	Fine motor	Self-help	Receptive language	Expressive language	Weight	Height	MUAC
Link	Logit	Logit	logit	identity (Latent)	identity	identity	Identity	Identity	identity	Identity	logit
**Primary outcome**											
** Infection in 0–59 days**	**-0.6(-1.2,-0.04)**	**-0.1(-1.0, 0.8)**	**-0.3(-1.2, 0.7)**	**0.02(-0.3, 0.3)**	**-1.3(-4.4, 1.7)**	**0.6(-1.2, 2.4)**	**-1.1(-3.7, 1.4)**	**-1.3(-3.5-1.0)**	**-0.2(-0.4, 0.01)**	**-0.2 (-0.4**,**-0.01)**	**0.6(-3.5, 1.0)**
**Demographic characteristics**											
** Gender**					**4.1(1.6, 6.6)**						
** Father’s education**									**0.1(0.1, 0.2)**	**-0.4(-0.6, -0.2)**	
** Mother’s education**					**3.0(1.5, 4.3)**					**0.1(0.04, 0.2)**	**-0.4(-0.7, -0.1)**
** Consanguineous marriage of parents**					**-2.2(-4.8, -0.4)**						
** Father’s employment status**						**-3.3(-6.1, -0.6)**					
** Number of siblings dead**					**-1.6(-2.8,-0.4)**						
**Socioeconomic characteristics**											
** Socioeconomic status**					**-3.9(-6.5, -1.3)**						
** Members in household**				**0.04 (0.01,0.1)**					**-0.5(-0.7, -0.3)**		
** Drinking water**				**-0.3(-0.6,-0.01)**						**-0.3(-0.4, -0.1)**	
** Fuel used for lighting**									**0.4(0.1, 0.8)**	**0.5(0.1, 0.8)**	
** Time to the nearest health facility**					**-0.2(-0.3, -0.1)**	**-0.1(-0.1,-0.01)**	**-0.2(-0.3, -0.1)**	**-0.1(-0.2,-0.02)**	**-0.01(-0.01,<-0.01)**		
** Ever admitted to school**		**1.1(0.2, 2.0)**	**1.0(0.1, 1.9)**	**-0.6(-0.9, -0.3)**	**17.5(14.3,20.6)**			**5.9(3.6, 7.0)**	**0.4(0.3, 0.6)**	**0.5(0.3, 0.7)**	**-0.9(-1.4, -0.3)**
** Can read/write any language**					**4.1 (0.8,7.5)**		**4.2 (2.0,6.4)**				
**Addiction/abuse/trauma**											
** Any mental trauma risk**				**0.7(0.4, 1.0)**							
** Addiction history of caregiver**					**-2,8(-5.3, -0.3)**						
** Physical abuse to participant**							**-3.8(-7.1 ,-0.1)**				
**Maternal/perinatal characteristics**											
** Place of birth**	**-0.7(-1.1, -0.2)**						**1.6(0.03, 3.2)**	**2.2(0.9, 3.6)**			
** Gestational age at birth**				**0.9(0.6, 1.2)**				**-2.1(-4.0, -0.3)**			
** Weight at birth**				**-0.6(-0.9, -0.2)**						**0.3(0.03, 0.5)**	
**Early nutritional characteristics**											
** Total breastfeeding duration**				**-0.02(-0.03,-0.01)**							
** Age at weaning**							**0.2(0.01, 0.5)**				
** MUAC**					**-5.4(-8.3, -2.4)**						

## Discussion

In our study, cases exposed to PSBI in early infancy performed poorly across various physical and neurodevelopmental domains assessed by the TQS, SDQ and PEDS:DM-AL tools at 6-9 years of age. We report several socioeconomic and environmental factors to be associated with neurodevelopment in this age group.

The most prominent abnormality in our population was seen in the social-emotional domain using the SDQ study tool. Previous studies utilizing the same tool have reported abnormal scores in the range of 22% to 34% among children and adolescents in Pakistan [16, 17]. The slightly higher prevalence of abnormality among cases in our study may be attributed to early infancy infections. This aligns with the Savioli et al. study, which identified the same as the second most prevalent neurodevelopmental abnormality in full-term babies affected by neonatal sepsis, and the most prevalent in suspected sepsis cases (cases displaying clinical signs of sepsis but no microbial growth, similar to our study population) [18]. A systematic analysis of Global Burden of Disease data from 1990 to 2015 ranked sensory disabilities fourth, conduct disorders seventh, and autism spectrum disorders ninth as leading causes of Years Lived with Disability (YLD) in the 5-9 years age group [19]. In our study, approximately 5% of mothers reported vision and hearing issues among the participants.

Our findings are consistent with long-term emotional-behavioral outcomes of infant survivors of invasive group B Streptococcus (iGBS) in 5 LMICs which reported increased anxiety, attention, and conduct problems for school-aged iGBS survivors compared with the non-iGBS group [20]. In the retrospective study by Savioli et al., children with confirmed and suspected neonatal sepsis cases had higher odds ratios of 1.48 and 1.09 reported for adverse neurodevelopmental outcomes. These ratios were adjusted for factors related to prematurity [18]. In addition, Savioli et al. also reported adjusted odds ratios ranging from 1.14 (communication domain in suspected sepsis cases) to over 4 (motor, learning, and autism spectrum disorder domains in known sepsis cases) [18]. Our results mirror these trends, with the highest issues observed in the motor domain, which showed a significant difference between cases and healthy controls. The motor development variables in our study included delayed motor milestones (TQS tool), current motor functions (TQS tool), and the fine motor neurodevelopmental domain (PEDS:DM-AL tool). We found all these scores to be lower among cases. 

Our study had several strengths, we were able to longitudinally track the ANISA birth cohort including and perform a standardized birth surveillance data collection and assessment of covariates. Our study tools, TQS and SDQ, are validated, widely accessible, and commonly used across Pakistan. They can serve as valuable screening tools, especially in resource-constrained settings. The ability to assess numerous covariates was facilitated by data available from birth records and baseline covariate forms. Limitations of our study include the absence of data from the upper-middle and upper socioeconomic classes. Since two-thirds of the pSBI cases were lost to follow-up due to migration out of the study area, we were unable to achieve the calculated sample size. To overcome the differences between the groups we carried out an age and gender matched analysis. Although the PEDS:DM-AL tool was culturally adapted, it had not been validated in the Pakistani population. Nonetheless, psychologists conducted the assessment and explained items to parents and participants using standardized methods as per tool instructions. Another inherent limitation lies in Pakistan's high infant and child mortality rates, which remove some severely affected individuals from the pool of survivors. Notably, our sample exhibited a similar trend.

Pakistan has a lack of resources for the timely identification and treatment of neurodevelopmental delays in children and adolescents. Early Childhood Care and Education (ECCE) remains a private sector engagement, largely inaccessible to the economically disadvantaged, particularly those in low-income areas [21]. The treatment gap for developmental disorders in rural Pakistan is nearly 100% [21]. Early interventions have proven to be scalable, and cost-effective not only in healthcare but also in education and social domains [22]. The implementation of transdiagnostic, task-shifting intervention strategies within primary healthcare settings supervised by existing specialist healthcare facilities involving training parents and caregivers to administer evidence-based interventions for developmental disorders emerges as a potential solution. Our findings can help inform government-initiated programs, for integrated maternal and child healthcare (iMNCH) such as the parental package, developed in partnership with UNICEF, which includes essential resources such as training manuals for parents, counselling cards, and educational materials, reaching over 17,000 families through multi-sector training initiatives and extensive social media and radio outreach [21]. The integration of this package into existing systems, coupled with our study findings on clinical predictors, enhances its impact on child and adolescent mental health in Pakistan. The package has been currently integrated into the existing system: Newborn Care Package, IMNCI curriculum, Health Policy Punjab, LHW curriculum Sindh, EPI-IPC (Expanded program on Immunization-Infection Prevention & Control) manual in Khyber Pakhtunkhwa, and IPC manual Polio [21]. Future steps involve finalizing implementation plans, integrating ECD (Early Childhood Development) modules into technology-based training, and advancing an integrated ECD-sensitive approach within the health, nutrition, and education sectors for sustainable impact.

## Conclusion

Our study showed significant associations between early infancy infections and delayed physical and developmental milestones particularly in the motor and social-emotional domains. It reveals the interplay of health, socio-economic, and environmental factors in shaping child development outcomes in low-resource settings. In addition, time to reach nearby health facilities, home deliveries, tap water consumption, school attendance, birth weight, breastfeeding duration, caregiver addiction history and socioeconomic status were found to negatively influence child development scores. Whereas mother's education, school admission, and literacy skills had positive impacts on fine motor skills underscoring the multifaceted nature of developmental outcomes.

### Supplementary Information

**Supplementary Material 1.**

## Data Availability

The dataset for this manuscript will be available from the corresponding author upon request.
