# Supplementary material for: Neurodevelopmental outcomes following possible serious bacterial infection in early infancy in Karachi, Pakistan: a prospective cohort study
Source: BMC Pediatr. 2024 May 15;24:336. doi: 10.1186/s12887-024-04780-5 (PMC11094884; doi:10.1186/s12887-024-04780-5)
Supplement: Supplementary file 1 — Supplementary Material 1. [file 12887_2024_4780_MOESM1_ESM.docx]

**Figure S1** The Final model retains all the pathways from the base model and all significant pathways from variables.

real_casecontrol

binomial

logit

SDQ

?

1

1.1

**SDQ_emotional**

Gaussian

identity

1.9

?

2

2.8

**sdq_conduct**

Gaussian

identity

2

?

3

1.9

**sdq_hyper**

Gaussian

identity

1.8

?

4

2.2

sdq_peer

Gaussian

identity

.93

?

5

1.4

sex

binomial

logit

**TQS Milestones**

binomial

logit

3

**TQS Vision**

binomial

logit

2.4

**TQS Hearing**

binomial

logit

2.6

**WAZ**

Gaussian

identity

-1.8

E

6

.89

**HAZ**

Gaussian

identity

-1.7

E

7

.95

cousinmarriage

binomial

logit

delay_fm

Gaussian

identity

75

?

8

217

delay_sh

Gaussian

identity

93

?

9

78

delay_rl

Gaussian

identity

95

?

10

155

delay_el

Gaussian

identity

82

?

11

119

sdq_prosocial

Gaussian

identity

9.2

?

12

1.9

**MUAC**

ordinal

logit

edum

ordinal

logit

eduf

ordinal

logit

mt_yes

binomial

logit

sib_dead

Gaussian

identity

Ever_school

binomial

logit

Read_write

binomial

logit

earn_dad

binomial

logit

members

Gaussian

identity

SES_bin

binomial

logit

fuel_light

binomial

logit

time_clinic

Gaussian

identity

**Drinking Water**

binomial

logit

add_adult

binomial

logit

physical_abuse

binomial

logit

birth_place

binomial

logit

gest_age

binomial

logit

birth_wt

binomial

logit

bf3

Gaussian

identity

wean

Gaussian

identity

.017

.71

-.63

.04

-.29

.95

-.57

-.019

1

1.1

1

.54

-.62

-.65

-.1

1.1

-.25

.98

-.19

-.49

.14

.44

.42

-.0072

-.2

-.43

.59

.14

.47

.47

-.25

.27

-1.3

4.1

-2.2

57

78

82

-5.4

3

-1.6

17

4.1

-3.9

-.21

-2.8

.63

59

52

-3.3

-.08

-1.1

80

4.2

-.18

-3.8

1.6

.23

-1.3

5.3

-.11

2.2

-2.1

-.32

.55

-.4

-.85

Please note that the predictor variables have been colored according to their respective sections. Grey-shaded areas represent variables from the same outcome tools or category.

| ***Color** | **Group of predictors** |
| --- | --- |
| Light green | Primary exposure |
| Yellow | Demographic |
| Grey | Socioeconomic |
| Teal | Addiction/abuse/trauma |
| Pink | Maternal/perinatal |
| Blue | Early nutrition |

Table Predictors associated with primary exposure (Early infancy infections)
